# Low- to high-density lipoprotein cholesterol ratio followed by coronary computed tomography angiography improves coronary plaque classification accuracy

**DOI:** 10.18632/oncotarget.23558

**Published:** 2017-12-21

**Authors:** Xiyang Hu, Wei Zhang, Nairui Zhao, Rongcheng Zhao, Shuofeng Li

**Affiliations:** ^1^ Department of Radiology, Cangzhou Central Hospital, Hebei, 061000, Cangzhou, China; ^2^ Department of Radiology, Cangzhou Hospital of Integrated Traditional and Western Medicine, Hebei, 061000, Cangzhou, China; ^3^ Department of Endocrinology, Cangzhou Central Hospital, Hebei, 061000, Cangzhou, China; ^4^ Department of Cardiology, Cangzhou Central Hospital, Hebei, 061000, Cangzhou, China

**Keywords:** noncalcified plaque, mixed plaque, coronary computed tomography angiography, low- to high-density lipoprotein cholesterol ratio, intravascular ultrasound

## Abstract

Coronary computed tomography angiography (CCTA) is a noninvasive test for detection and analysis of coronary plaques morphology and classification. The low- to high-density lipoprotein cholesterol (L/H) ratio is associated with plaques vulnerability. The study aims to investigate the diagnostic accuracy of CCTA and L/H ratio for plaques classification. We enrolled 212 patients with coronary artery single-vessel disease who performed preoperative CCTA and Intravascular ultrasound (IVUS)-guided invasive coronary angiography. Patients were assigned to the acute coronary syndrome (ACS) group (*n =* 129) and stable angina pectoris (SAP) group (*n =* 83). CCTA showed that patients with ACS had more soft plaque and less calcific plaque than those with SAP. The plaque volume and remodeling index measured by CCTA showed good correlation with those measured by IVUS. IVUS identified 91 soft, 58 mixed and 63 calcific plaques in this cohort. For diagnosis of noncalcified plaque (soft and mixed), CCTA had the sensitivity and specificity of 87.9% and 90.4%, respectively. While refer to the further diagnosis of mixed plaque from noncalcified plaque, the sensitivity and specificity was 88.4% and 88.8%, respectively. The L/H ratio was gradually decreased from soft plaque to calcific plaque. If the patients had both the two characteristics (L/H ≥ 2.55 and CCTA), the sensitivity, and specificity were improved in diagnosing noncalcified plaque or mixed plaque. In conclusion, a combined application of CCTA and L/H ratio improves the diagnostic accuracy for coronary noncalcified plaque or mixed plaque as compared to CCTA along.

## INTRODUCTION

Major adverse cardiovascular events are often the initial manifestation of coronary artery disease (CAD), which is related mostly to one pathophysiologic substrate: the rupture or erosion of a vulnerable plaque [1]. Intravascular ultrasound (IVUS) is capable of cross-sectional imaging of coronary arteries and a comprehensive assessment of coronary atherosclerotic plaques. However, due to its invasiveness, the related increased risk during the procedure, and high cost, IVUS cannot be used for routine evaluation of plaque characteristics [2]. Meanwhile, recent improvements in computed tomography (CT) technology and the advent of the multislice CT have spurred interest in noninvasive detection of morphologic characteristics of coronary plaques using coronary CT angiography (CCTA). Identification of noncalcified plaques, particularly mixed plaques, has been considered as important features of plaque vulnerability and instability [3–5]. Plenty of studies have shown CCTA is capable to identify several morphologic and geometric characteristics of atherosclerotic plaques and demonstrated a good correlation between plaque composition derived from CCTA and IVUS [6–8]. Nevertheless, for diagnosis of noncalcified plaque, CCTA has relative low sensitivity and specificity than calcified plaques [9] because of the substantial overlap of the density values between lipid-rich and fibrous plaques [10].

Since biomarkers are proposed for risk stratification due to their relative ease of use, high-sensitivity cardiac troponin T (hs-cTnT) [11–13], NT-proBNP [14], Cystatin C [15], serum matrix metalloproteinase-9 [16], and lipoprotein-associated phospholipase A2 [17] have been considered closely related with coronary plaque morphology. Of note, a recent study revealed that the low-density lipoprotein-cholesterol/high-density lipoprotein-cholesterol (L/H) ratio independently had a significant fixed effect with the percentage of the lipid component of coronary plaques [18] and a high L/H ratio was reported closely associated with larger numbers of low density noncalcified plaque [19]. Hence, there are expectations that with the assistance of L/H ratio, the diagnostic accuracy for noncalcified plaque (especially the mixed one) in patients with CAD of CCTA would significant increase. On above data, the aim of our study was to assess the diagnostic performance of CCTA and L/H ratio by using invasive IVUS as the reference standard.

## RESULTS

### Clinical characteristics and laboratory results of study subjects

**Figure 1:**
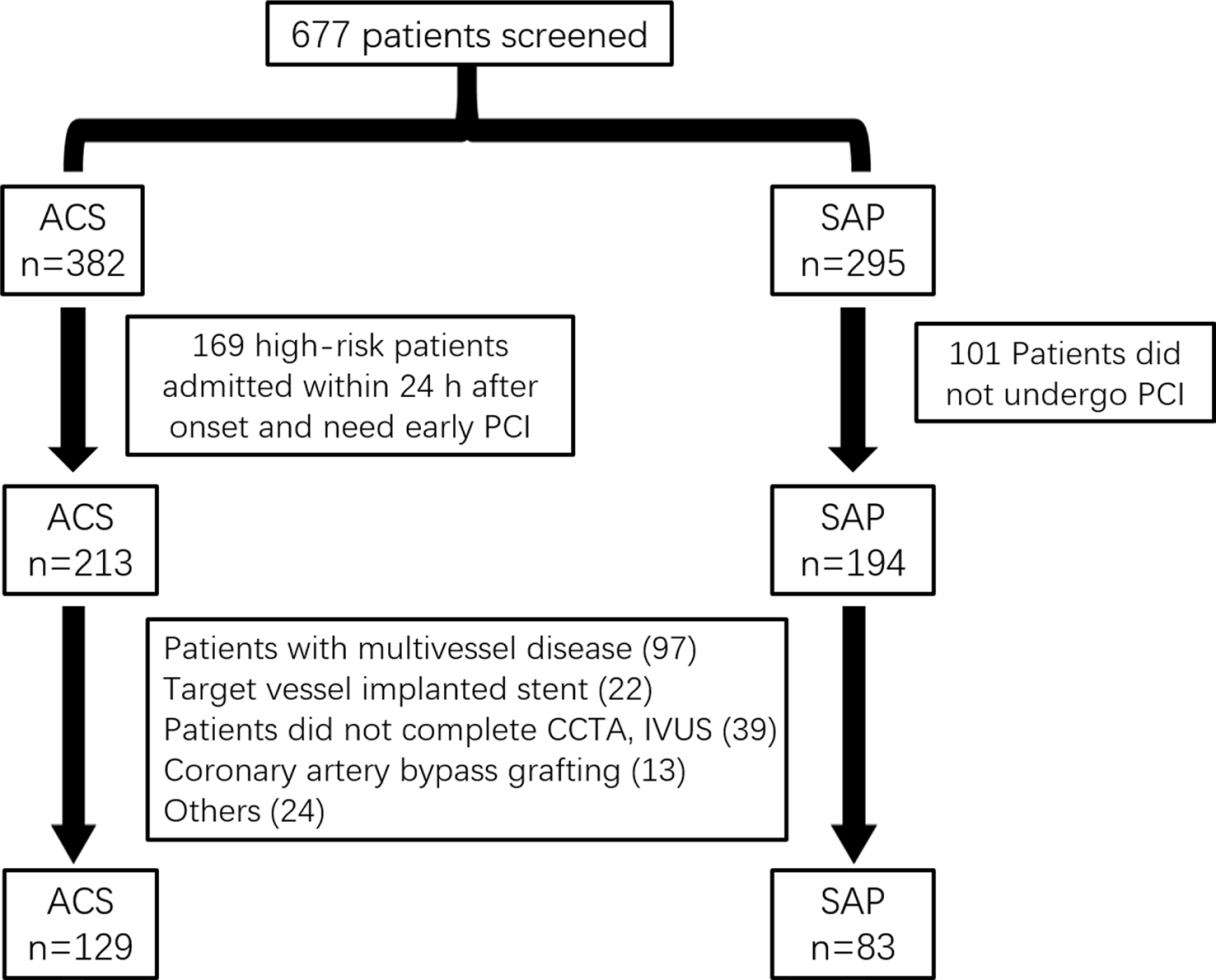
Flow diagram of the phases of the study

The procedures followed in this study were depicted in Figure 1. The final 212 participants that enrolled in this study comprised 119 men and 93 women with a mean age of 54.3 ± 11.9 years. As shown in Table 1, there was no statistical differences between ACS and SAP group when comparing to the age, sex, BMI, current smoking, hypertension and diabetes mellitus. The level of hs-cTn T and NT-proBNP were significant higher in the ACS group when compared to the SAP group (*P* < 0.05). Use of beta-blockers, ACEI/ARB, calcium channel blocker, diuretic and statins were equal between the two groups. Although no significant differences were observed in HDL-C or LDL-C levels between groups, L/H ratio differed significantly between two groups (*P* < 0.001). We also identified that patients with ACS had higher levels of creatinine, hs-CRP and Hcy than those with SAP (*P* < 0.05 for all).

**Table 1:** Demographic, clinical characteristics and laboratory results of the study groups

Clinical Characteristics	ACS group (*n =* 129)	SAP group (*n =* 83)	*P* value
Age (years)	54.2 ± 12.1	55.4 ± 10.8	0.463
Male (n, %)	77(59.7)	42 (50.6)	0.246
BMI (kg/m2)	28.1 ± 3.70	27.6 ± 4.41	0.374
Current smoking (*n*, %)	62 (48.1)	33 (39.8)	0.296
Hypertension (*n*, %)	80 (62.0)	43 (51.8)	0.184
Diabetes mellitus (*n*, %)	64 (49.6)	37 (44.6)	0.565
Medications			
Beta-blockers	90 (69.8)	59 (71.1)	0.792
ACEI/ARB	112 (86.8)	73 (88.0)	0.976
Calcium channel blocker	42 (32.6)	30 (36.1)	0.697
Diuretic	68 (52.7)	37 (44.6)	0.310
Statins	118 (91.5)	71 (85.5)	0.259
Laboratory tests			
Hs-cTn T (ng/L)	7.80 ±3.26	4.43 ±2.91	< 0.001
D-dimer (μg/mL)	3.41±1.77	3.12 ±1.69	0.237
NT-proBNP (pg/ml)	283 ± 180	232 ± 119	0.024
Triglyceride (mg/dl)	1.67 ± 0.73	1.56 ± 0.46	0.222
HDL-C (mmol/L)	1.19 ± 0.37	1.31 ± 0.49	0.058
LDL-C (mmol/L)	3.17 ± 1.38	2.89 ± 0.92	0.077
L/H ratio	2.53 ± 0.63	2.21 ± 0.54	< 0.001
HbA1c (%)	6.13 ± 1.39	5.83 ± 0.91	0.083
Scr (μmol/L)	79.1 ± 18.3	72.5 ± 13.5	0.005
Hs-CRP (mg/L)	5.81 ± 2.12	3.93 ± 1.87	< 0.001
Hcy (μmol/L)	11.2 ± 5.65	7.69 ± 3.73	< 0.001

Note: BMI = body mass index; hs-cTn T = high sensitive cardiac Troponin T; HDL-C = high-density lipoprotein cholesterol; LDL-C = low-density lipoprotein cholesterol; L/H=LDL-C/HDL-C; Scr = Serum creatinine; Hs-CRP = high-sensitivity C-reactive protein; Hcy = homocysteine.

### CCTA and IVUS characteristics of coronary plaques

The culprit lesion of each subject was determined during ICA. Coronary angiography revealed 212 lesions, containing 159 Left anterior descending artery (LAD, 75%), 20 Left circumflex artery (LCX, 9.4%), and 33 Right coronary artery (RCA,15.6%). As illustrated in Table 2, lesion length, calcium score, vessel volume, lumen volume and minimum lumen area (MLA), showed no significant differences between two groups (*P* > 0.05 for all). CCTA showed that although, plaque volume was higher in the ACS group than in the SAP group, no significant difference was observed (*P* = 0.083). In addition, noncalcified plaque volume and plaque burden were significant higher, while the remodeling index was lower in ACS group than that in SAP group (126 ± 78.3 vs. 101 ± 66.1 mm^3^, *P* = 0.017; 72.7 ± 9.88 vs. 68.3 ± 8.35, *P* = 0.001; 1.05 ± 0.36 vs. 1.31 ± 0.49, *P* < 0.001). Plaques were defined as soft (*n* = 82), mixed (*n* = 55) and calcific (*n* = 75) according to the CCTA findings. Patients in ACS group owned more soft plaque and less calcific plaque than those with SAP (*P* = 0.030).

**Table 2:** CCTA measures characteristics

CCTA Parameter	ACS group (*n =* 129)	SAP group (*n =* 83)	*P* values
Lesion length, mm	16.5 ± 12.0	18.5 ± 12.6	0.247
Calcium score	390 (107–975)	311 (115–723)	0.410
Vessel volume (mm3)	406 ± 255	396 ± 227	0.772
Plaque volume (mm3)	169 ± 107	144 ± 93.6	0.083
NCPV (mm3)	126 ± 78.3	101 ± 66.1	0.017
Lumen volume (mm3)	237 ± 133	252 ± 149	0.446
MLA (mm2)	2.51 ± 1.70	2.96 ± 1.72	0.063
Plaque burden (%)	72.7 ± 9.88	68.3 ± 8.35	0.001
Remodeling index	1.05 ± 0.36	1.31 ± 0.49	< 0.001
Plaque morphology*			0.030
Soft (*n =* 82)	53 (41.1)	29 (34.9)	
Mixed (*n =* 55)	39 (30.2)	16 (19.3)	
Calcific (*n =* 75)	37 (28.7)	38 (45.8)	

Note: NCPV=noncalcified plaque volume; MLA = minimum lumen area. ^*^ obtained from CCTA.

### The accuracy of CCTA in evaluating plaque characteristic

**Figure 2:**
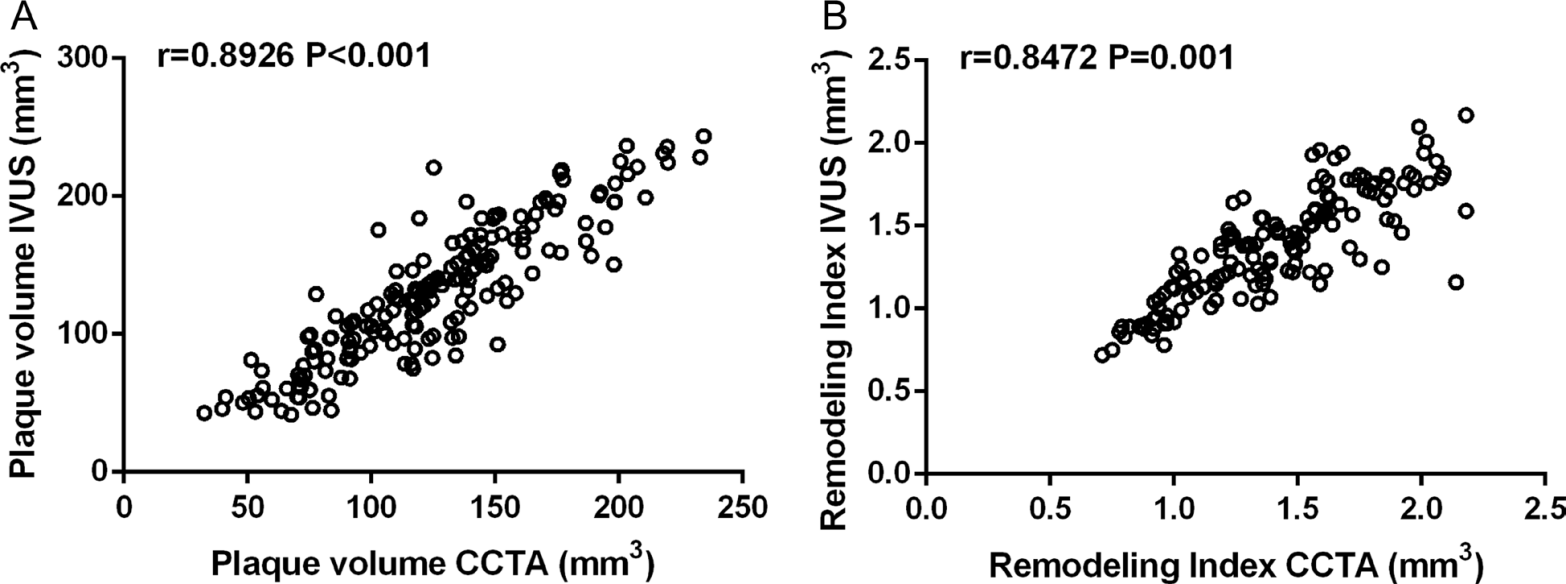
(**A**) Univariate linear correlation analysis showed the correlation of plaque volume in intravascular ultrasound (IVUS) and coronary computed tomography angiography (CCTA) (*n* = 212, r = 0.8926, *P* < 0.001). (**B**) Correlation of remodeling index in IVUS and CCTA (*n* = 212, r = 0.8472, *P* < 0.001).

**Figure 3:**
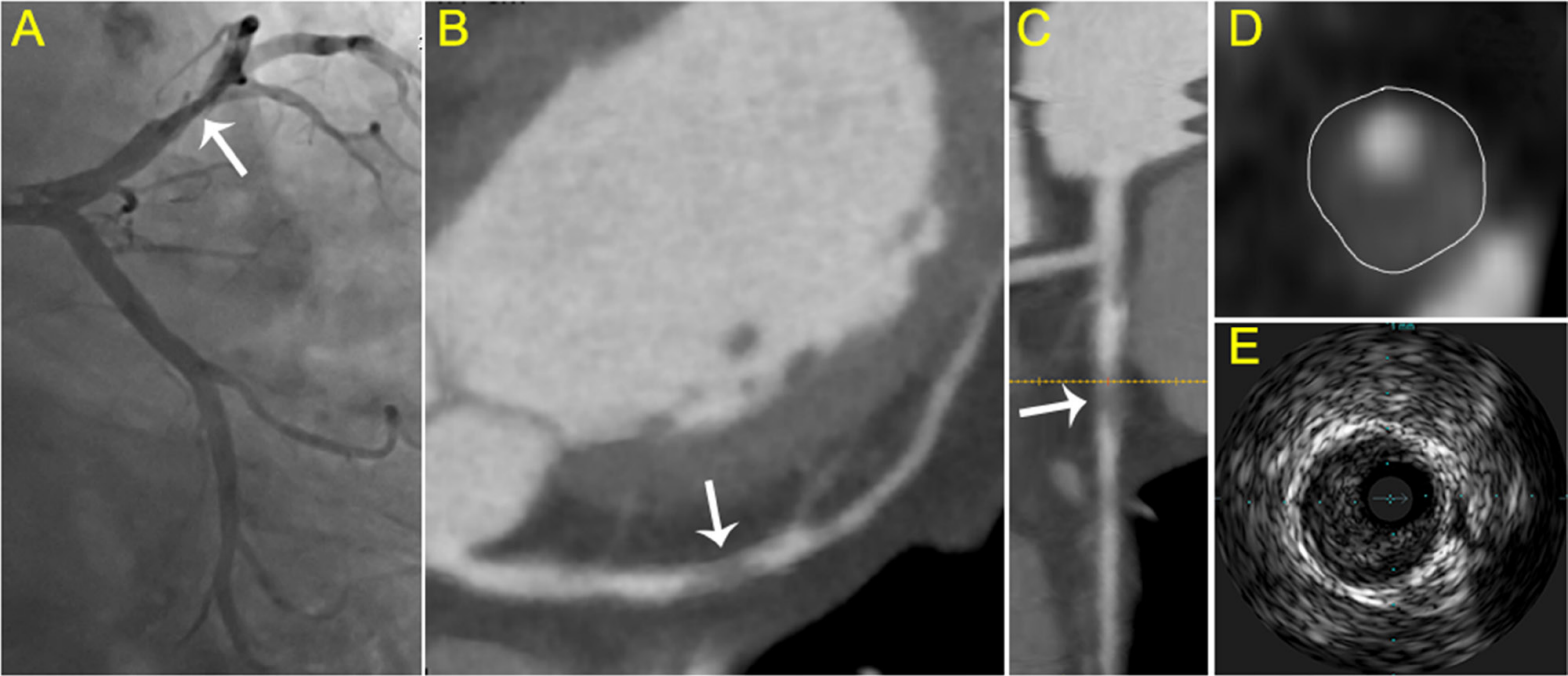
Typical visualization of soft plaque (**A**) Angiography showed the culprit vessel in the proximal LCX. (**B**) axial view and (**C**) curved planar reformat of CCTA shows relevant plaque causing stenosis of the LCX (arrow). (**D**) cross-sectional view of soft plaque with low attenuation. (**E**) corresponding IVUS images.

**Figure 4:**
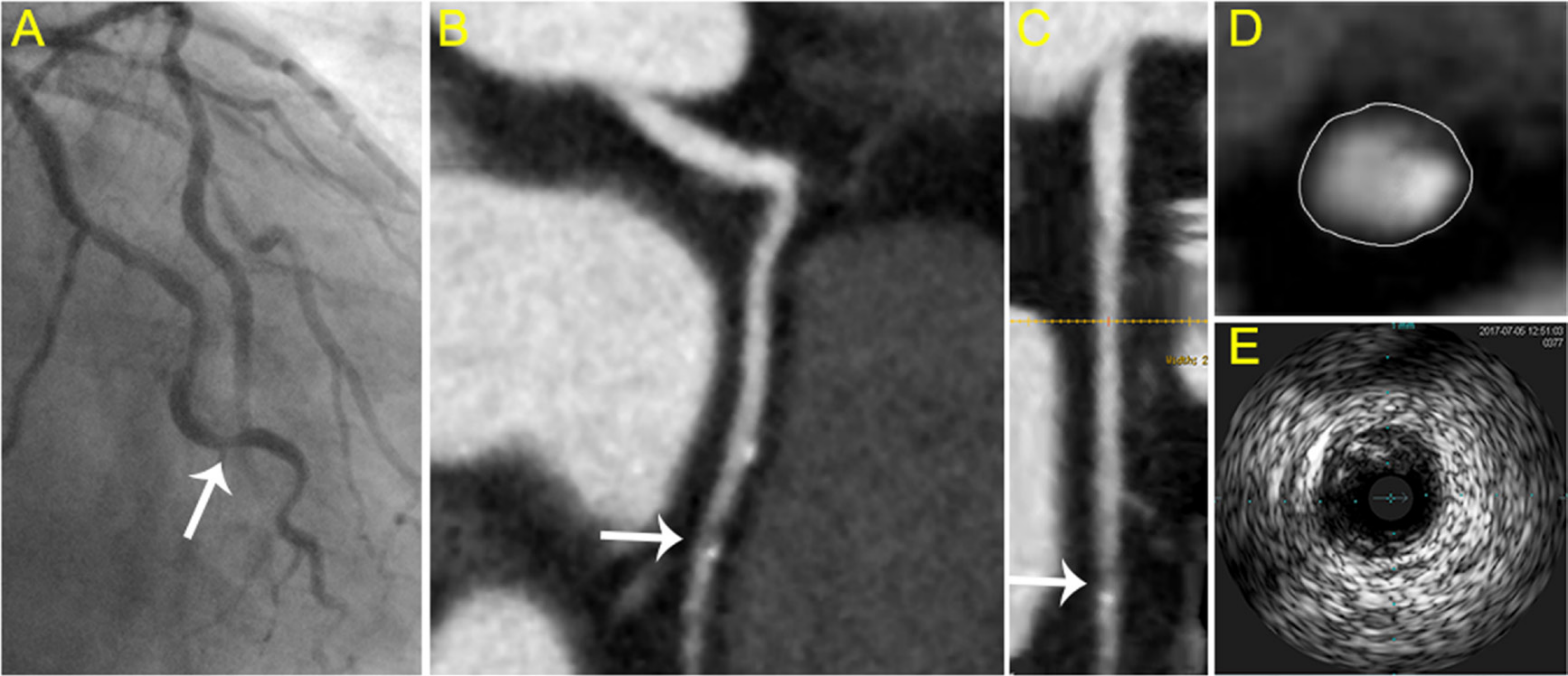
Typical visualization of mixed plaque (**A**) Angiography showed the culprit vessel in the proximal LAD. (**B**) axial view and (**C**) curved planar reformat of CCTA shows relevant plaque of the LAD (arrow). (**D**) cross-sectional view of mixed plaque with eccentricity spotty calcification. (**E**) corresponding IVUS images.

**Figure 5:**
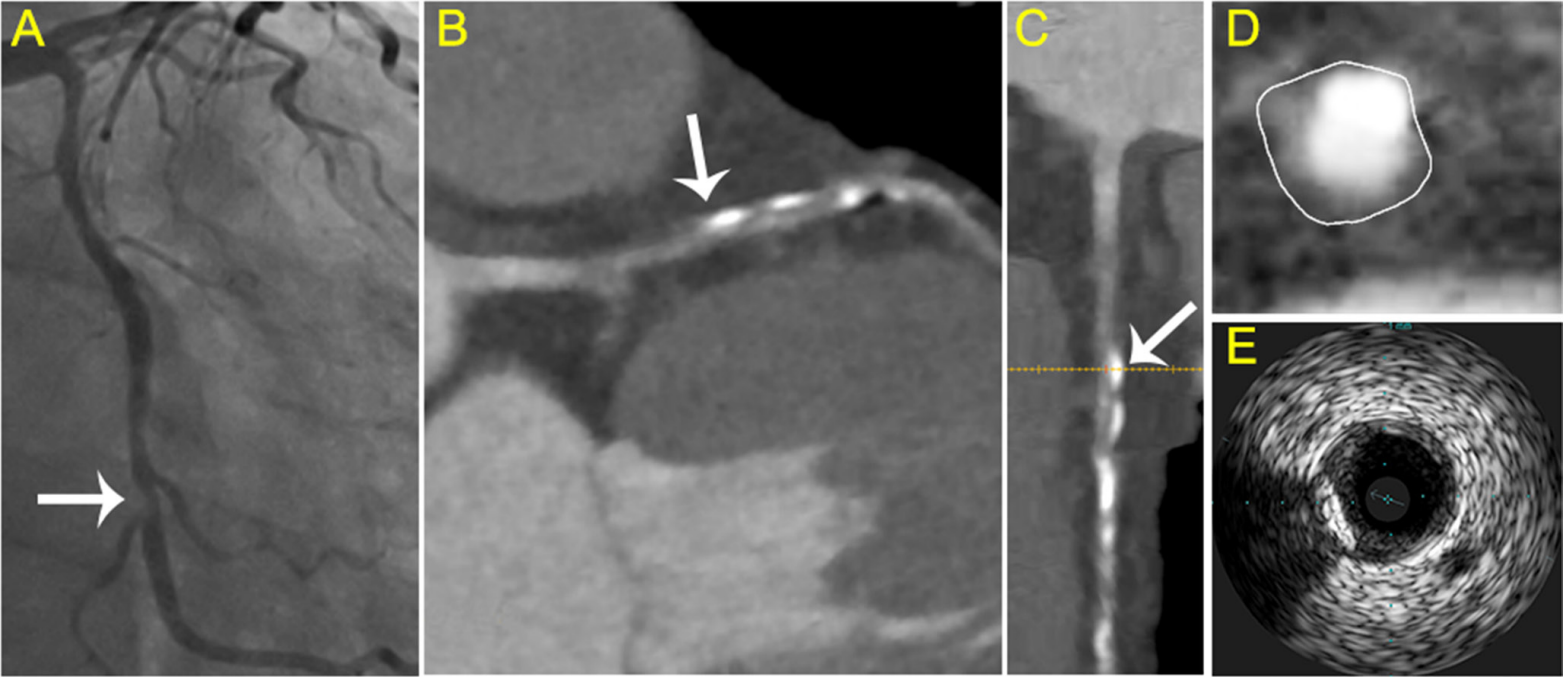
Typical visualization of calcified plaque (**A**) Angiography revealed the culprit vessel in the middle LAD. (**B**) axial view and (**C**) curved planar reformat of CCTA shows relevant plaque of the LAD (arrow). (**D**) cross-sectional view of calcified plaque with calcification in the side portion. (**E**) corresponding IVUS images.

The mean plaque volume detected by IVUS were 155 ± 119 mm^3^. Pearson’s correlation coefficient was r = 0.8926 (*P* < 0.001) when analyzed the correlation of plaque volume derived from CCTA and IVUS (Figure 2A). In the 212 lesions investigated by CCTA and IVUS, the mean Remodeling Index was 1.22 ± 0.46 in CCTA and 1.27 ± 0.35 in IVUS (r = 0.8472, *P* < 0.001, Figure 2B). IVUS identified 91 soft, 58 mixed and 63 calcific plaque in total. The plaque subtypes classified by CCTA gave close agreement with that evaluated by IVUS (Table 3). In general, the plaque subtypes were misclassified by 128-slice CT in 39 of 212 atherosclerotic lesions. For diagnosis of noncalcified plaque (soft and mixed), the sensitivity, specificity, positive predictive value (PPV) and negative predictive value (NPV) was 87.9%, 90.4%, 95.5% and 76.0%, respectively. While refer to the further diagnosis of mixed plaque from noncalcified plaque, the sensitivity, specificity, PPV and NPV was 88.4%, 88.8%, 83.8% and 80.0%, respectively (Table 4). Examples of CCTA plaque types and corresponding IVUS imaging are presented in Figures 3–5.

**Table 3:** Consensus table of 128-slice CT and IVUS to detect and classify coronary plaques

	IVUS		
128-slice CT	Soft	Mixed	Calcific
Soft	76	5	1
Mixed	10	40	5
Calcific	5	13	57

**Table 4:** Diagnostic accuracy data for CCTA in detecting coronary plaque

	Sensitivity (%)	Specificity (%)	Positive Predictive Value (%)	Negative Predictive Value (%)	Accuracy (%)
Noncalcified	87.9	90.4	95.5	76.0	88.7
mixed	88.4	88.8	83.8	80.0	88.5

Note: noncalcified included soft and mixed plaque.

### Association of L/H ratio with the characteristics of coronary plaque

As CCTA had a relative low power to identified noncalcified plaque, we further compared the expression levels of laboratory values between different plaque subtypes, in order to provide cool idea to improve the diagnostic value of CCTA. Unexpected, Table 5 showed that the levels of hs-cTn T and LDL-C differed in plaque classification and L/H ratio showed the most change among groups (*P* < 0.001). Thereby, we investigate the different clinical parameter between groups with high or low L/H ratio. We found that patients with L/H ≥ 2.55 were older and had higher BMI, compared to those with L/H < 2.55 (Table 6). Subsequent multivariate regression analyses revealed that independent predictors of L/H ≥ 2.55 were age (β = 1.019, 95% CI: 0.012–2.239, *p* = 0.044), BMI (β = 1.158, 95% CI: 0.144 to 4.903, *p* = 0.022), and Hcy (β = 1.428, 95% CI: 0.107–4.748, *p* = 0.010) (Table 7).

**Table 5:** The comparison analysis of various serum biomarkers in different classified plaques

Biomarkers	Soft (*n =* 91)	Mixed (*n =* 58)	Calcific (*n =* 63)	*P* value
hs-cTn T (ng/L)	6.37 ±3.25	5.77 ±2.67	4.73 ±2.41^*^	0.003
D-dimer (μg/mL)	3.14 ±1.55	3.27 ±1.32	3.66±1.80	0.126
NT-proBNP (pg/ml)	271 ± 197	246 ± 141	229 ± 129	0.287
HDL-C (mmol/L)	1.20 ± 0.34	1.26 ± 0.37	1.12 ± 0.29	0.068
LDL-C (mmol/L)	3.20 ± 1.58	2.82 ± 1.32	2.60 ± 1.12^*^	0.027
L/H ratio	2.78 ± 0.56	2.45 ± 0.60*	2.39 ± 0.71^#*^	< 0.001
Scr (μmol/L)	71.8 ± 13.9	73.6 ± 14.2	75.4 ± 17.5	0.351
Hs-CRP (mg/L)	5.06 ± 2.47	4.89 ± 1.72	4.33 ± 2.07	0.115
Hcy (μmol/L)	10.3 ± 3.28	9.73 ± 4.63	8.92 ± 4.16	0.109

Note: hs-cTn T = high sensitive cardiac Troponin T; HDL-C = high-density lipoprotein cholesterol; LDL-C = low-density lipoprotein cholesterol; L/H = LDL-C/HDL-C; Scr = Serum creatinine; Hs-CRP = high-sensitivity C-reactive protein; Hcy = homocysteine. ^*^P < 0.05 vs. Soft group, ^#^*P* < 0.05 vs. Mixed group obtained from one-way ANOVA followed by least significant difference (LSD) multiple comparison test.

**Table 6:** Patient characteristics stratified by L/H ratio

Parameter	L/H < 2.55 *n =* 89	L/H ≥ 2.55 *n =* 123	*P* values
Age (years)	52.1 ± 9.5	54.9 ± 10.2	0.043
Male (*n*, %)	48 (53.9)	79(64.2)	0.172
BMI (kg/m2)	26.9 ± 3.75	28.2 ± 3.96	0.017
Hypertension (*n*, %)	54 (60.7)	69 (56.1)	0.599
Diabetes mellitus (*n*, %)	36 (40.4)	63 (51.2)	0.158
Hyperlipidemia (*n*, %)	49 (55.1)	73 (59.3)	0.183
ACS (*n*, %)	51 (57.3)	78 (63.4)	0.099
STEMI	15 (16.9)	28 (22.8)	
NSTEMI	24 (26.9)	36 (29.2)	
Unstable Angina	12 (13.5)	14 (11.4)	

Note: HDL-C = high-density lipoprotein cholesterol; LDL-C = low-density lipoprotein cholesterol; L/H = LDL-C/HDL-C; BMI = body mass index; ACS = acute coronary syndrome; STEMI = ST segment elevation myocardial infarction; NSTEMI = non-ST segment elevation myocardial infarction.

**Table 7:** Multivariate linear regression analyses between L/H ratio and clinical parameters

Marker	Standardized correlation coefficient	95%CI	*P*
Age (years)	1.019	0.012, 2.239	0.044
BMI (kg/m^2^)	1.158	0.144, 4.903	0.022
Hypertension	–0.967	–1.145, 3.149	0.208
Diabetes mellitus	1.157	–0.546, 2.986	0.349
Triglyceride (mg/dl)	0.045	−0.031, 0.248	0.133
HbA1c (%)	0.038	–0.014, 0.188	0.405
Scr (μmol/L)	–1.417	–3.127, 4.506	0.551
Hs-CRP (mg/L)	0.088	–0.002, 0.175	0.069
Hcy (μmol/L)	1.428	0.107, 4.748	0.010

Note: CI = confidence interval; Scr = Serum creatinine; Hs-CRP = high-sensitivity C-reactive protein; Hcy = homocysteine.

### Diagnostic value for coronary plaque subtypes by combining CCTA, and L/H ratio

The sensitivity and specificity would be 89.9% and 79.3% when L/H alone was used to identify noncalcified plaque. Interesting, when combining with CCTA, the sensitivity, specificity, PPV and NPV increased to 95.9%, 93.6%, 97.3% and 90.8% for diagnosing noncalcified plaque respectively. Moreover, if L/H ratio was used to recognize mixed plaque from noncalcified plaque, the sensitivity, specificity, PPV and NPV would be 86.0%, 91.7%, 94.8% and 78.6%. If the patients had both the two characteristics (L/H ≥ 2.55 with CCTA), the sensitivity, specificity, PPV and NPV would be 92.1%, 94.4%, 96.5% and 87.9% for diagnosis of mixed plaque respectively. All the sensitivity, specificity, PPV, NPV for plaque classification according to the L/H ratio and CCTA were shown in Table 8.

**Table 8:** Diagnostic value for coronary plaque subtypes by combining CCTA, and L /H

Category	Sensitivity (%)	Specificity (%)	Positive Predictive Value (%)	Negative Predictive Value (%)	Accuracy (%)
Noncalcified plaque					
L/H ratio	89.9	79.3	91.2	76.9	86.8
CCTA + L/H ratio	95.9	93.6	97.3	90.8	95.3
Mixed plaque					
L/H ratio	86.0	91.7	94.8	78.6	88.1
CCTA + L/H ratio	92.1	94.4	96.5	87.9	93.0

Note: noncalcified included soft and mixed plaque.

## DISCUSSION

The accurate characterisation of coronary atherosclerotic plaques remains challenging. Limited spatial and contrast resolutions of most clinical imaging techniques prevent classifications that exactly match histology. Currently, classifications provided by IVUS or optical coherence tomography are probably the closest to histopathology [20, 21]. We assessed the diagnostic performance of CCTA or L/H ratio or both, comparison with IVUS in the current study. The detection of mixed plaque is of potential relevance owing to their propensity to rupture and cause future cardiac events [22]. The noncalcified plaques, thereby, were further divide into soft and mixed plaques in the present study because thin-cap fibroatheromas were most prevalent in mixed plaques [8].

To confirm the accuracy of 128-slice CT in evaluating plaque morphology, we found plaque volume and remodeling index measured by CCTA were accurate when compared with those measured by IVUS, which was similar with previous studies [6, 23]. We then compared to quantitative functional markers derived from CT and IVUS and found good correlation in plaque volume and remolding index. This phenomenon was also observed in the studies by Achenbach et al. [6] and Voros et al. [24] We identified 63 calcific plaque and 149 noncalcified plaques using IVUS in our cohort. When using contrast enhanced 128-slice CT, the accuracy was 88.7%, and the sensitivity and specificity was 87.9% and 90.4%. The results were similar with a meta-analysis by Gao et al. that CT has higher diagnostic sensitivity and specificity for calcified plaques (93% and 98%) than noncalcified plaques (88% and 92%) [25]. CT is known for its ability to show calcifications, but visualization of intraplaque hemorrhage (homogeneous plaques without calcification are stable fibrous plaques) is difficult [26]. For distinguishing soft and mixed plaque, the results are also not promising resulting from significant overlap in attenuation values between individual plaques [23, 25, 27]. The sensitivity and specificity was 88.4% and 88.8%, respectively in this study for diagnosing mixed plaques.

Although CCTA offers an interesting option to noninvasively detect, quantify and characterize coronary plaque, the above diagnostic accuracy is not sufficient for clinical application. So far, variety of risk factors, such as hypertension, diabetes, smoking and dyslipidemia are strongly associated with the development of CAD [28]. L/H ratio, rather than individual assessment of LDL-C and HDL-C, is more closely associated with the development of CAD [29] and accordingly, we also found age, BMI and the levels of Hcy had correlation with L/H ratio in patients with ACS and SAP. In addition, it is receiving considerable attention as a new risk factor for coronary plaque vulnerability. A meta-analysis suggested that a decrease of L/H ratio after statin treatment was significant associated with regression of the atheroma volume [30]. In the current study, we found L/H ratio differed significantly between different plaque type determined by IVUS. The ratio was highest in the soft plaque and lowest in the calcific plaque. These results may be explained by a study about evaluation the lipid component of non-culprit lesions that the L/H ratio was the most relevant parameter in predicting the lipid component of coronary plaques [18], as the lipid component is gradually decreased from soft plaque to calcific plaque. Another study also identified that high L/H ratio was associated with a high percentage of lipid volume and a low percentage of fibrous volume in left main coronary artery lesions [31]. Besides, investigators found an inverse correlation between LDL-C level and the minimum plaque CT density. An L /H > 2.5 could predict low-density plaques (OR 2.39, 95%CI: 1.28–4.86) [19]. And similarly, a high L/H ratio was a useful marker for unstable plaques in patients with SAP [32]. We for the first time identified the L/H ≥ 2.55 enables characterization of mixed plaque with an acceptable sensitivity (86.0%) and specificity (91.7%) both in patient with ACS and SAP. These finding triggered us to combined it with CCTA to improve the diagnostic accuracy. A combination of L/H ≥ 2.55 and CCTA yield an excellent sensitivity and PPV for diagnosing noncalcified plaque and a high specificity and PPV for mixed plaque. Based on the foregoing data, we conclude that the joint application of L/H ratio and CCTA is useful in classifying coronary plaque types. It is no doubt that L/H could not replace the imaging tools for identification plaque types, but may act as a complementary method. Improvement in imaging techniques may take years, but this is really an easy obtained biomarker.

Certain limitations of our study should be mentioned. Firstly, we could not analyze L/H ratio in subgroup with normal, low or high HDL-C levels, which may lead to the overestimate of its diagnostic accuracy. Some patients with genetically induced low levels of HDL-C may also influence L/H ratio. As statins treatment could decrease plaque volume [33] and L/H ratio [34], whether the change of L/H ratio during the lipid-lowering therapy could greatly affect its diagnostic value is still unclear. We await future studies to systematically assess the distribution of L/H ratio in patients with CAD. Moreover, we only identified the diagnostic value of L/H ratio and CCTA in patients with single-vessel disease. It is still unknown whether L/H ratio could help improve CCTA’s diagnostic accuracy in those with multi-vessel disease. Importantly, there is a designed observational cohort study, we only accessed the value of L/H ratio or CCTA in plaque classification but not evaluate their property in predicting ACS. Prospective studies with following up are needed to further confirm the value of combination of L/H ratio and CCTA before its use in the clinical settings.

## MATERIALS AND METHODS

### Study design and population

From March 2014 to June 2016, patients admitted to the emergency department of Cangzhou Central Hospital were screened. They were matched for age, sex, body mass index (BMI), cardiovascular risks, and medications used. All the enrolled patients had blood collection, CCTA examination and IVUS imaging. Culprit lesion was determined during non-emergent invasive coronary angiography (ICA). Exclusion criteria of the study were (1) patients with multivessel disease (2) infectious endocarditis, congenital heart disease, cardiomyopathy, and primary valvular disease; (3) culprit lesion has implanted stent, had a history of coronary artery bypass grafting; (4) symptomatic peripheral vascular disease, thyroid disease, autoimmune disease, hematologic disease, malignant tumor or other severe disease; (5) did not complete the CCTA or IVUS imaging; (6) did not offer informed consent.

All subjects met the criteria were categorized into SAP group (*n* = 83) and ACS group (*n* = 129). The SAP group was classified based on the guidelines of the American College of Cardiology (ACC)/American Heart Association (AHA) [35]. Patients with single-vessel disease who were suggested to have elective PCI were enrolled in this study. These patients underwent CCTA examination before non-emergent ICA. Patients with ACS were identified in accordance with the guidelines of the ACC/AHA [36]. The ACS group included 43 patients with ST-segment elevation myocardial infarction (STEM), 60 patients with non–ST-segment elevation myocardial infarction (NSTEM), and 26 patients with unstable angina. High-risk patients admitted within 24 h after onset were excluded because these patients needed early ICA and PCI. Thereby, those presenting 24 h after onset of chest pain or intermediate- and low-risk patients were studied.

Finally, a total of 212 were admitted (Figure 1). The study was approved by the Research Ethics Committee of the Cangzhou Central Hospital, and informed consent was obtained from all individuals.

### Coronary computed tomographic angiography

All subjects underwent CCTA before conventional invasive coronary angiography (ICA). We performed electrocardiographic-gated CTA using a 128-slice multi detector computed tomography CT scanner (Somatom Definition AS+; Siemens Medical Solutions, Forchheim, Germany) with a 2 × 64 ×0.6-mm slice collimation, a gantry rotation time of 0.35 s and a tube voltage of 120 kV, and the tube modulation was from 300 to 420 mA, depending on the patient’s height and weight. Patients with a heart rate of ≥ 60 beats/min received 20 mg of oral metoprolol 60 min before scanning and all patients received 0.3mg nitroglycerin sublingually before CT examination. Imaging was achieved using a contrast-enhanced ECG gating scanner. During CCTA acquisition, patients received 1.2 ml/kg body weight of iodinated contrast (Iohexol 350 mg I/ml, GE Healthcare, UK) at a rate of 3.0 ml/s followed by 40 ml saline solution at a rate of 3.0 ml/s. The arterial phase was obtained with a delay time of 35 s, portal venous phase with 70 s and equilibrium phase with 3 min. The raw CT data were reconstructed using algorithms optimized for retrospective ECG-gated segment reconstruction with a 0.6-mm slice thickness and a 0.3 mm increment. Other characteristics were previously described [37].

### Quantitative analysis of CCTA

The target vessel was the culprit lesions. A cardiologist and a radiologist, who were blinded to the patients’ clinical characteristics, analyzed the CCTA data, with maximum intensity and curved multiplanar reconstruction techniques. We calculated total calcium score and expressed as Agatston score [38]. A dedicated semi-automatic software prototype (Plaque Analysis 2.0.3 FRONTIER, Siemens) was used for the plaque analysis. All lesions with ≥ 25% stenosis on coronary CTA were evaluated. The start and end of a culprit lesion was defined as the proximal and distal non-diseased section with absence of atherosclerotic changes. CTA measures included lesion length, minimum lumen area (MLA), vessel volume, plaque volume, noncalcified plaque volume, and lumen volume. Plaque burden%= (plaque area/vessel area) × 100. The remodeling index was obtained by dividing the vessel diameter at the plaque site by the diameter at reference segment. Plaque morphology on CCTA was characterized as a) soft, b) mixed, c) calcified. Atheroma with density greater than 150 hounsfield unit (HU) and lesions with 50% or greater calcium was considered calcific plaque. Noncalcified plaques were subdivided into soft plaques with lipid core with a cutoff point of lower than 30 HU and mixed plaques (30–150 HU) [39].

### Invasive coronary angiography and intravascular ultrasound

Selective coronary angiography was performed in multipleorthogonal views using standard techniques within two weeks after CCTA and images were stored digitally. Angiograms were evaluated by two cardiologists blinded to both the purpose of the research and the subjects’ CTA results. Angiographic plaque morphology, Thrombolysis In Myocardial Infarction (TIMI) grading, the severity of luminal stenosis were analyzed according to established invasive criteria [40, 41] and culprit lesions were confirmed according to CAG, location of asynergy in Electrocardiograph, and location of ST-segment elevation in STEMI. When referring to SAP, the lesions > 50%, with the evidence of the subsequent IVUS, were defined as culprit lesions.

IVUS examination was performed after 200 μg of intracoronary nitroglycerin immediately after guidewire crossing or dilation with a 1.5- to 2.5-mm balloon, to examine the culprit lesion. The IVUS images were achieved using an iLab Ultrasound Imaging system (Boston Scientific Corp, Natick, MA, USA) and a 40 MHz 2.5 Fr intravascular catheter. The catheter was advanced greater than 10mm beyond the plaque, with the assistance of an automatic pullback device at 0.5 mm/s. Images were quantified for external elastic membrane area (EEM), lumen and plaque cross-sectional area and plaque volume. Volumes were calculated using Simpson’s rule and also reported as normalized area (volume divided by length) [42]. Plaques were classified as a) soft, b) mixed, c) calcified, on the basis of the clinical expert consensus document on IVUS acquisition, measurement and reporting [43]. Soft plaque was defined as plaque tissue producing echogenicity less than that of the surrounding adventitia, in the absence of any calcium. mixed plaque was defined as atheroma having density equal or little more than to that of the adventitia without acoustic shadowing. Calcific plaque was defined as atheroma brighter than the adventitia with acoustic shadowing.

### Blood tests

Blood samples were collected from all subjects prior to CCTA, the samples were drawn into tubes with ethylene diamine tetraacetic acid (EDTA) or sodium citrate and were separated by centrifugation at 3,000 rpm for 10 minutes within 2 hours. Then the samples were divided into three tubes, with immediate storage at -80°C until analysis. ELECSYS 2010 automated analyser (Roche Diagnostics, Mannheim, Germany) was used to detect the level of hs-Tn T [44]. The level of D-dimer, NT-proBNP, Triglyceride, LDL, HDL, glycated hemoglobin (HbA1c), Serum creatinine (Scr), high-sensitivity C-reactive protein (hs-CRP) and homocysteine (Hcy) were measured using a clinical automated biochemistry analyzer (Cobas6000; Roche Diagnostics).

### Statistical analysis

The data were analyzed using SPSS version 22.0 (SPSS Inc., Chicago, IL, USA). Continuous variables were presented as medians ± standard deviation and categorical variables were expressed as frequencies with percentages. The normality test of each variable was conducted by using the Kolmogorov-Smirnov test. Differences between groups were evaluated by Student’s *t*-test or chi-square analysis. One-way analysis of variance (ANOVA) was used to compare the difference between more than two groups, and the differences between groups were subsequently determined by least significant difference (LSD) test when appropriate. A Kruskal-Wallis test was used for non-normally distributed data. Pearson’s correlation coefficients were calculated between plaque volume, as well as Remodeling index, obtained from CCTA or IVUS. Patients were divided into groups according to the median of L/H ratio (2.55). The sensitivity, specificity, PPV, NPV, and diagnostic accuracy of L/H ratio alone or in combination CCTA were calculated. Multivariable analysis was performed to determine independent predictors for L/H ≥ 2.5 including age, BMI, hypertension, diabetes mellitus, triglyceride, HbA1c, Scr, hs-CRP, and Hcy. A *P*-value of < 0.05 was considered statistically significant.

## CONCLUSIONS

In conclusion, combined L/H ratio and CCTA improves the diagnostic accuracy for noncalcified plaques as compared to CCTA along by using invasive IVUS as the reference standard. This has the potential to improve risk stratification and is useful for the subsequent treatment decisions.
